# Supplementary material for: Asymbiotic germination and morphological studies of seeds of Atlantic Rainforest micro-orchids (Pleurothallidinae)
Source: PLoS One. 2020 Dec 14;15(12):e0243297. doi: 10.1371/journal.pone.0243297 (PMC7735581; doi:10.1371/journal.pone.0243297)
Supplement: Attachment — Submitted filename: PONE-D-20-27111_reviewer jcc.pdf [file pone.0243297.s002.pdf]

PLOS ONE

Asymbiotic germination and morphological studies of seeds of Atlantic Rainforest micro-orchids (Pleurothallidinae)

--Manuscript Draft--

**Manuscript Number:**	PONE-D-20-27111
**Article Type:**	Research Article
**Full Title:**	Asymbiotic germination and morphological studies of seeds of Atlantic Rainforest micro-orchids (Pleurothallidinae)
**Short Title:**	Asymbiotic germination and morphological studies of micro-orchids
**Corresponding Author:**	Luciana Lopes Fortes Ribas, Dra<br>Federal University of Parana: Universidade Federal do Parana<br>Curitiba, Paraná BRAZIL
**Keywords:**	Orchidaceae; micromorphology; in vitro germination; woody plant medium
**Abstract:**	<p class="Corpo" style="text-align:justify;text-indent:35.45pt;line-height:200%">We studied the morphological and morphometric characters of seeds belonging to 11 species of the subtribe Pleurothallidinae using light and scanning electron microscopy to understand the <i>in vitro</i> germination process. The viability of the seeds was evaluated by <i>in vitro</i> germination in woody plant medium (WPM), and by analysis of the developmental stages of protocorms until seedling formation (two to 24 weeks). Morphometric data showed variations within the genus <i>Acianthera </i>and between species of different genera. The best germination and protocorm formation responses occurred with <i>Acianthera prolifera </i>(92%) and <i>Acianthera ochreata</i> (86%), with the formation of seedlings after 12 and 16 weeks of sowing, respectively. The seeds and embryos of these species were larger (length, width, and volume) than the others, with a structural polarity that may have facilitated their germination. Other characteristics of <i>A. prolifera</i> seeds that may have contributed to these results include the presence of a thin testa without ornamentation and a suspensor. The protocorms of <i>Anathalis</i> <i>obovata, Dryadella liliputiana</i>, and <i>Octomeria gracillis</i> developed slowly in the WPM, not reaching the seedling stage in 24 weeks of cultivation. This morphological and morphometric study contributes to the understanding of asymbiotic germination of some micro-orchid species. <o:p></o:p></p>
**Order of Authors:**	Franciele Marx Koene
	Érika Amano
	Eric de Camargo Smidt
	Luciana Lopes Fortes Ribas, Dra
**Additional Information:**	
**Question**	**Response**
**Financial Disclosure**<br><br>Enter a financial disclosure statement that describes the sources of funding for the work included in this submission. Review the submission guidelines for detailed requirements. View published research articles from *PLOS ONE* for specific examples.	The author(s) received no specific funding for this work.

Powered by Editorial Manager® and ProduXion Manager® from Aries Systems Corporation

This statement is required for submission and **will appear in the published article** if the submission is accepted. Please make sure it is accurate.<br><br>**Unfunded studies**<br>Enter: *The author(s) received no specific funding for this work.*<br><br>**Funded studies**<br>Enter a statement with the following details:<br>• Initials of the authors who received each award<br>• Grant numbers awarded to each author<br>• The full name of each funder<br>• URL of each funder website<br>• Did the sponsors or funders play any role in the study design, data collection and analysis, decision to publish, or preparation of the manuscript?<br>• **NO** - Include this sentence at the end of your statement: *The funders had no role in study design, data collection and analysis, decision to publish, or preparation of the manuscript.*<br>• **YES** - Specify the role(s) played.<br><br>* typeset	
**Competing Interests**<br><br>Use the instructions below to enter a competing interest statement for this submission. On behalf of all authors, disclose any competing interests that could be perceived to bias this work—acknowledging all financial support and any other relevant financial or non-financial competing interests.<br><br>This statement **will appear in the published article** if the submission is accepted. Please make sure it is accurate. View published research articles from *PLOS ONE* for specific examples.	NO authors have competing interests

Powered by Editorial Manager® and ProduXion Manager® from Aries Systems Corporation

**NO authors have competing interests**<br><br>Enter: *The authors have declared that no competing interests exist.*<br><br>**Authors with competing interests**<br><br>Enter competing interest details beginning with this statement:<br><br>*I have read the journal's policy and the authors of this manuscript have the following competing interests: [insert competing interests here]*<br><br>* typeset	
**Ethics Statement**<br><br>Enter an ethics statement for this submission. This statement is required if the study involved:<br><br>• Human participants<br>• Human specimens or tissue<br>• Vertebrate animals or cephalopods<br>• Vertebrate embryos or tissues<br>• Field research<br><br>Write "N/A" if the submission does not require an ethics statement.<br><br>General guidance is provided below. Consult the submission guidelines for detailed instructions. **Make sure that all information entered here is included in the Methods section of the manuscript.**	N/A

**Format for specific study types**<br><br>**Human Subject Research (involving human participants and/or tissue)**<br>• Give the name of the institutional review board or ethics committee that approved the study<br>• Include the approval number and/or a statement indicating approval of this research<br>• Indicate the form of consent obtained (written/oral) or the reason that consent was not obtained (e.g. the data were analyzed anonymously)<br><br>**Animal Research (involving vertebrate animals, embryos or tissues)**<br>• Provide the name of the Institutional Animal Care and Use Committee (IACUC) or other relevant ethics board that reviewed the study protocol, and indicate whether they approved this research or granted a formal waiver of ethical approval<br>• Include an approval number if one was obtained<br>• If the study involved *non-human primates*, add *additional details* about animal welfare and steps taken to ameliorate suffering<br>• If anesthesia, euthanasia, or any kind of animal sacrifice is part of the study, include briefly which substances and/or methods were applied<br><br>**Field Research**<br><br>Include the following details if this study involves the collection of plant, animal, or other materials from a natural setting:<br>• Field permit number<br>• Name of the institution or relevant body that granted permission	
**Data Availability**<br><br>Authors are required to make all data underlying the findings described fully available, without restriction, and from the time of publication. PLOS allows rare exceptions to address legal and ethical concerns. See the PLOS Data Policy and FAQ for detailed information.	Yes - all data are fully available without restriction

Powered by Editorial Manager® and ProduXion Manager® from Aries Systems Corporation

A Data Availability Statement describing where the data can be found is required at submission. Your answers to this question constitute the Data Availability Statement and **will be published in the article**, if accepted.<br><br>**Important:** Stating 'data available on request from the author' is not sufficient. If your data are only available upon request, select 'No' for the first question and explain your exceptional situation in the text box.<br><br>Do the authors confirm that all data underlying the findings described in their manuscript are fully available without restriction?	
**Describe where the data may be found in full sentences. If you are copying our sample text, replace any instances of XXX with the appropriate details.**<br><br>• If the data are **held or will be held in a public repository**, include URLs, accession numbers or DOIs. If this information will only be available after acceptance, indicate this by ticking the box below. For example: *All XXX files are available from the XXX database (accession number(s) XXX, XXX.).*<br>• If the data are all contained **within the manuscript and/or Supporting Information files**, enter the following: *All relevant data are within the manuscript and its Supporting Information files.*<br>• If neither of these applies but you are able to provide **details of access elsewhere**, with or without limitations, please do so. For example:<br><br>*Data cannot be shared publicly because of [XXX]. Data are available from the XXX Institutional Data Access / Ethics Committee (contact via XXX) for researchers who meet the criteria for access to confidential data.*<br><br>*The data underlying the results presented in the study are available from (include the name of the third party*	All relevant data are within the manuscript and its Supporting Information files.

and contact information or URL).<br>• This text is appropriate if the data are owned by a third party and authors do not have permission to share the data.<br><br>* typeset	
Additional data availability information:	Tick here if the URLs/accession numbers/DOIs will be available only after acceptance of the manuscript for publication so that we can ensure their inclusion before publication.

Powered by Editorial Manager® and ProduXion Manager® from Aries Systems Corporation

Manuscript
Click here to access/download;Manuscript;manuscriptPlos.docx

Asymbiotic germination and morphological studies of seeds of Atlantic Rainforest micro-orchids (Pleurothallidinae)

Asymbiotic germination and morphological studies of micro-orchids

Franciele Marx Koene[1], Érika Amano[1], Eric de Camargo Smidt[1] and Luciana Lopes Fortes Ribas[1*]

[1] Programa de Pós-Graduação em Botânica , Setor de Ciências Biológicas, Universidade Federal do Paraná, Curitiba, PR, Brazil

* Corresponding author

E-mail: llfribas@ufpr.br (L.L.F. Ribas)

Abstract

We studied the morphological and morphometric characters of seeds belonging to 11 species of the subtribe Pleurothallidinae using light and scanning electron microscopy to understand the *in vitro* germination process. The viability of the seeds was evaluated by *in vitro* germination in woody plant medium (WPM), and by analysis of the developmental stages of protocorms until seedling formation (two to 24 weeks). Morphometric data showed variations within the genus *Acianthera* and between species of different genera. The best germination and protocorm formation responses occurred with *Acianthera prolifera* (92%) and *Acianthera ochreata* (86%), with the formation of seedlings after 12 and 16 weeks of sowing, respectively. The seeds and embryos of these species were larger (length, width, and volume) than the others, with a structural polarity that may have facilitated their germination. Other characteristics of *A. prolifera* seeds that may have contributed to these results include the presence of a thin testa without ornamentation and a suspensor. The protocorms of *Anathalis obovata*, *Dryadella liliputiana*, and *Octomeria gracillis* developed slowly in the WPM, not reaching the seedling stage in 24 weeks of cultivation. This morphological and morphometric study contributes to the understanding of asymbiotic germination of some micro-orchid species.

Introduction

Orchids are suffering from an uncertain future through overexploitation, habitat loss due to human activities and the impact of climate change, and their survival is contingent on a variety of abiotic and biotic factors and their effect on orchid growth, development, and reproduction [6]. Their unusual physiology, seed structure, and germination pattern set them apart from other flowering plants. The seeds are dust-like, non-endospermic, and require a fungal stimulus for germination in nature [5, 39]. A unique characteristic of orchid seeds is that, rather than an endosperm,the air space surrounds a tiny globular embryo containing a small number of cells, which is protected by a membranous testa; the air-space volume in orchid seeds varies depending on the species [13, 42]. Seed morphological traits are thus related to biological and ecological processes like dormancy, germination, and seed dispersal [13].

Orchid propagation in nature is highly complex, involving specific mycorrhizal associations. *In vitro* methods, or plant tissue culture, can provide alternative approaches for both propagation and preservation, with asymbiotic germination representing an ideal system for studying the growth and development of seeds and seedlings [20, 31]. *In vitro* germination of orchids can help increase the effectiveness of conservation and breeding programs due to their high germination rates, which are commonly over 70%, as opposed to under 5% in *ex vitro* conditions [10].

The Orchidaceae is one of the largest and most diverse families of flowering plants in the world, with 25,000–28,000 species [5]. One of the subtribes of this family, Pleurothallidinae Lindl., belongs to Epidendroideae subfamily and Epidendreae tribe, consisting of approximately 5,100 species known as micro-orchids [19]. They are found in the Neotropics, from Argentina to southern Mexico. Micro-orchids in the Atlantic Forest in Brazil, which is home to a large number of micro-orchid species and is one of

the most threatened extinction biomes on the planet, face extreme extinction pressures with a reduction in the original habitat of over 90% [26, 30, 33]. Most species of Pleurothallidinae have no great commercial appeal, due to the small dimensions of the plants and their flowers, as well as the difficulties of cultivation [26]. There are few studies of asymbiotic germination of micro-orchids, which have shown low germination rates and slow growth of seedlings [14, 27]. Studies on the morphology and morphometry of the seeds and their relationship with micro-orchid germination are also scarce.

Although the seeds of different orchid species have many similarities, there is significant variability in the size, shape, characteristics of the testa cells, the zones of adhesion, and sculptures constituted by the cellular wall and cuticular material [34]. Studies on seed morphology and morphometry have achieved substantial contributions to the taxonomy, phylogeny, and phytogeography of this group [1, 7]. Meanwhile, these studies can also enhance understanding of the *in vitro* germination process, which in turn can accelerate the production of seedlings for future reintroduction into their natural habitat, aiding in the conservation of the Pleurothallidinae. Our study was carried out with 11 native species belonging to six genera of the Atlantic Forest that have not been evaluated for the threat of extinction by the *Flora of Brazil* [8, 9], as follows: *Acianthera aphthosa* (Lindl.) Pridgeon & M. W. Chase, *Acianthera hatschbachii* (Schltr.) Chiron & van den Berg, *Acianthera ochreata* (Lindl.) Pridgeon & M.W. Chase, *Acianthera prolifera* (Herb. ex Lindl.) Pridgeon & M. W. Chase, *Acianthera sonderiana* (Rchb.f.) Pridgeon & M.W. Chase, *Anathallis obovata* (Lindl.) Pridgeon & M.W. Chase, *Dryadella lilliputiana* (Coan.) Luer, *Dryadella zebrina* (Porsch) Luer, *Octomeria gracilis* Lodd. ex Lindl., *Pabstiella fusca* (Lindl.) Chiron & Xim. Bols, and *Stelis grandiflora* Lindl. *Acianthera aphthos*a was the only species

considered critically endangered (CR) in the Brazilian state of Espírito Santo by the Red List [35]. Five of these species are endemic to Brazil (*A. ochreata*, *A. aphthosa*, *A. sonderiana*, *D. liliputiana*, and *O. gracilis*) [8, 9]. *Acianthera prolifera* is rupicolous and *A. ochreata* is rupicolous and epiphytic, while all the other species in this study are epiphytes.

This study aimed to analyze the morphological and morphometric characteristics of the seeds and relate them with the asymbiotic germination of species of the subtribe Pleurothallidinae. We demonstrate efficient methods of rapid propagation of some species and our results offer real possibilities for reintroduction programs that can play a key role in reducing the extinction risk for species of micro-orchids.

Materials and Methods

Seed materials

Mature seeds of 11 species of the Pleurothallidinae subtribe (Fig 1A) were collected from naturally dehiscing capsules (Fig 1B) from individuals in a greenhouse of the Federal University of Paraná (UFPR), Curitiba, Paraná, Brazil. Table 1 shows the list of voucher specimens deposited at the Herbarium of the Botany Department, UFPR, and the seeds used for the morphological and morphometric studies for all species, and for asymbiotic germination.

Table 1. Species of the subtribe Pleurothallidinae used for morphological, morphometric, and *in vitro* germination analysis in this study.

Species	Voucher	*In vitro* germination
Acianthera aphthosa (Lindl.) Pridgeon & M. W. Chase	Koene, FM. 010	X
A. hatschbachii (Schltr.) Chiron & van den Berg	Oliveira, LRL. 019	-
A. ochreata (Lindl.) Pridgeon & M.W. Chase	Koene, FM. 005	X
A. prolifera (Herb. ex Lindl.) Pridgeon & M. W. Chase	Koene, FM. 001	X
A. sonderiana (Rchb.f.) Pridgeon & M.W. Chase	Koene FM. 009	-
*Anathallis obovata (*Lindl.) Pridgeon & M.W. Chase	Santos, MC. 020	X
Dryadella liliputiana (Coan.) Luer	Imig, DC. 381	X
D. zebrina (Porsch) Luer	Imig, DC. 405	-
Octomeria gracilis Lodd. ex Lindl.	Koene, FM. 014	X
Pabstiella fusca (Lindl.) Chiron & Xim. Bols	Koene, FM. 013	X
Stelis grandiflora Lindl.	Ignowski, H. 011	X

Morphological and morphometric analysis of seeds

An average of 30 seeds per specimen was analyzed using a light (Fig 1C) and a scanning electron microscope (SEM). For SEM observations, the seeds were adhered with double-sided carbon tape and coated with gold. Qualitative data on the general seed morphology, including color, ornamentation, shape, micropillar opening, and the presence of a cuticular deposit, were analyzed. Quantitative analyses were performed using a light microscope (Olympus BX41 with DC30 camera), and the following variables were measured for seeds and embryos: length (L), width (W), L/W ratio, volume, and percentage of air space between the testa and the embryo. The width and

length were measured with a micrometer at the longest and widest axis of the seed. Seed volume was calculated as $2\,[(W/2)^2 \cdot (L/2) \cdot (\pi/3)]$. Embryo volume was calculated by using the formula $4/3\ \pi \cdot L/2 \cdot (W/2)^2$. The percentage of air space was calculated as [(seed volume - embryo volume) / seed volume] x 100 [1, 2].

Seed viability: tetrazolium test and *in vitro* germination

Seeds (5 mg) were placed in a 1.5 mL Eppendorf microtube and pre-conditioned in a 10% sucrose solution at room temperature for 24 h. They were then immersed in a 1% tetrazolium solution for 24 h at 40°C in a water bath in the dark. The solution was drained from the tubes with a micropipette, and the seeds were washed twice with distilled water, following the methodology proposed by Hosomi et al. [18]. Red seeds were classified as viable and used for morphometric studies.

Seeds were surface-sterilized by dipping into a 1% sodium hypochlorite solution (NaClO) (v / v) containing 0.1% Tween 20® (v / v) for 15 min while stirring. The seeds were then transferred to a funnel coated with sterile filter paper and washed six times with sterile distilled water. The seeds were dried on sterile filter paper and inoculated in Petri dishes (150 mm x 20 mm) containing 30 mL of the woody plant medium (WPM) [25]. The media were supplemented with 5.6 g L^{-1} agar from HiMedia® (Mumbai, India) and 3% sucrose (w/v). The pH of the media was adjusted to 5.8 with 0.1 N NaOH or HCl before the addition of agar. Culture media were sterilized by autoclaving for 20 min at 120°C.

About 500 seeds per Petri dish, with three Petri dishes per species, were inoculated. Protocorm development was evaluated from two to 16 weeks based on the following stages: 1, seed with chlorophyllous embryo (Fig 1D); 2, testa ruptured/chlorophyllous protocorm (germination) (Fig 1E); 3, protocorm with apex

and/or rhizoids (Fig 1F); 4, protocorm with one or two leaves; 5, protocorm with two or more leaves and root (seedling) (Fig 1G). The germination rate was evaluated after four, eight, and 12 weeks of cultivation, and the average time (in days) to reach the stages was calculated for 24 weeks. The Petri dishes with seeds were maintained at 26±2°C/18±2°C (day/night), with a 16 h photoperiod under fluorescent lamps at a light intensity of 40 µmol m^{-2} s^{-1}.

Fig 1. ***Acianthera ochreata.*** (A) Plant cultivated in the greenhouse, (B) dehiscent capsule, (C) seed observed in optical microscope, *in vitro* germination (D-H), (D) seed with chlorophyllous embryo, (E) testa ruptured/ chlorophyllous protocorm (germination), (F) protocorm with apex and / or rhizoids, (G) protocorm with two or more leaves, (H) seedling. Bar: A= 2 cm, B= 0,5 cm, C= 100 µm, D-G= 200 µm, H= 1cm.

Statistical analysis

The experimental design was completely randomized. The data of the asymbiotic germination were submitted to the Bartlett and the Shapiro-Wilk normality test and analysis of variance (ANOVA). The means were compared by the Tukey test at a level of significance of 5%. The statistical program used was PAST 3.3 software.

Results

Morphological and morphometric analysis of seeds

The seeds of the orchids studied exhibited diversity in their shape, size, volume, and seed coat (ornamentation) (Tables 2 and 3), as well as in their embryo morphometry (Table 4).

Pale yellow seeds were observed in *A. prolifera*, *D. lilitipuana*, and *S. grandiflora*, while the seeds of the other species studied were brown (Table 2).

The seeds had several shapes, including fusiform, filiform, ellipsoid, and clavate (Figs 2A-2K). Generally, cells were shorter at either pole, while the medial cells of the seed coat were elongated. The chalazal pole of the seeds was closed, and the micropillar end was open (Fig 2F), except for *D. zebrina,* which was also closed (Table 2). The testa cells observed were transparent, longitudinally oriented, and oblong (Fig 2L), except in *A. prolifera*, in which they were hexagonal (Fig 2M). The ornamentation pattern of the testa cells of some species included papillae or verrucosities (Fig 2N), though these were absent from *A. prolifera*, *A. obovata*, *S. grandiflora* and *A. hatschbachii* (Fig 2O) (Table 2). The number of testa cells ranged from 2 to 14 on the longest axis and 3 to 12 on the widest axis, with the highest number in *A. prolifera* and *A. ochreata* (Table 3).

Fig 2. Seeds of Pleurothallidinae species observed in scanning electron microscopy:. variation of seeds shape (A-K), (A) *Acianthera prolifera,* (B) *A. hatschbachii*, (C) *A. ochreata*, (D) *A. aphtosa*, (E) *A. sonderiana*, (F) *Anathallis obovata*, (G) *D. lilitipuana*, (H) *Dryadella zebrina*, (I) *Pabstiella fusca*, (J) *Octomeria gracillis*, (K) *Stellis grandiflora,* (L) seed testa with oblong cells of *P. fusca ,* (M) seed

testa with hexagonal cells of *A. prolifera,* (N) testa cells of *D. zebrina* with ornamentation, (O) seed testa with smooth cells of *A. hatschbachii*. Bar: A-H= 50µm, I-O=100µm

Table 2. Morphological seed characteristics of species belonging to the subtribe Pleurothallidinae.

Species	Color	Shape	Testa cell	Micropillar opening	Ornamentation
Acianthera aphthosa	Pale Yellow	Fusiform	Oblong	Yes	Papillae
A. ochreata	Brown	Ellipsoid	Oblong	Yes	Verrucosities
A. hatschbachii	Brown	Filiform	Oblong	Yes	Papillae
A. prolifera	Pale Yellow	Fusiform	Hexagonal	Yes	Absent
A. sonderiana	Brown	Clavate	Oblong	Yes	Verrucosities
Anathallis obovata	Pale yellow	Ellipsoid	Oblong	Yes	Absent
Dryadella liliputiana	Brown	Ellipsoid	Oblong	Yes	Verrucosities
D. zebrine	Brown	Ellipsoid	Oblong	No	Verrucosities
Pabstiella fusca	Brown	Clavate	Oblong	Yes	Papillae

Octomeria gracilis	Brown	Fusiform	Oblong	Yes	Papillae
Stellis grandiflora	Pale yellow	Clavate	Oblong	yes	Absent

The seeds showed high diversity in their size (length, width, and volume) (Table 3), as well as in the size and number of cells constituting the embryo (Table 4), despite their microscopic nature. The seed length ranged from 187±31 µm (*P. fusca*) to 742±119 µm (*A. prolifera*), while the width ranged from 83±7 µm (*P. fusca*) to 159± 18 µm for *A. sonderiana* (Table 3). *A. prolifera* and *A. ochreata* had length-to-width ratios of 2.700 and 1.767, respectively, while the L/W ratio in *P. fusca* was 0.140 (Table 3). *A. prolifera* had the largest seed volume (3.540 mm^3 x 10 $^{-3}$) while the smallest was recorded in *P. fusca* (0.337 mm^3 x 10 $^{-3}$) (Table 3).

Table 3. Morphometric data of seeds of species belonging to the subtribe Pleurothallidinae.

Species	Length (µm)	Width (µm)	Length/width Ratio	Testa cells* length-width	Volume mm^3 x 10^{-3}
Acianthera aphthosa	513 ±24	115±21	1.075	9 – 12	1.401
A. hatschbachii	401 ±27	111±31	1.386	6 – 12	1.293

A. ochreata	473 ±24	139±18	1.768	10 – 12	2.392
A. prolifera	742±119	135±43	2.700	14 – 10	3.540
A. sonderiana	307±81	159± 18	1.120	3 – 6	2.032
Anathallis obovata	244±31	109±17	1.101	4 – 6	0.759
Dryadella liliputiana	265±42	146±20	1.125	2 – 5	1.479
D. zebrine	262±17	154±20	1.720	2 – 3	1.626
Octomeria gracilis	247±28	90±16	1.084	5 – 6	0.524
Pabstiella fusca	187±31	83±7	0.140	4 – 3	0.337
Stelis grandiflora	195±19	127±21	1.053	2 – 5	0.823

The values represent the means and standard deviations.

*Testa cells: measurements on the largest length and width of the testa of the seed.

The embryos were generally ellipsoidal and located at the center of the seed. Variation in length, width, and volume was also observed. The largest length was recorded for *A. prolifera* while *A. ochreata* had the largest width and volume, and *P. fusca* embryos had the smallest length, width, and volume (Table 4).

The largest percentage of air space was observed in *A. sonderiana* (87.83%), whereas the lowest percentage of air space was observed in *O. gracilis* (61.21%) and *P. fusca* (56.14%) (Table 4). The seed volume to embryo volume ratio was highest (8.2167) in *A. sonderiana*, followed by *D. zebrina* (5.9256), and *A. hatschbachii* (5.1990) and lowest in *P. fusca* (2.2785) (Table 4).

Table 4. Morphometric data of embryos of species belonging to the subtribe Pleurothallidinae.

Species	Length (µm)	Width (µm)	Volume (mm^3 x 10^{-3})	Air space (%)	SV/EV*
Acianthera aphthosa	186±21	73± 14	0.5147	74.42	2.7220
A. hatschbachii	97±15	70± 15	0.2487	80.77	5.1990
A. ochreata	175±23	99±14	0.8976	62.48	2.6729
A. prolifera	216±47	80±9	0.7235	79.56	4.8929
A. sonderiana	84± 7	75±18	0.2473	87.83	8.2167
Anathallis obovata	76±17	69±6	0.1894	75.04	4.0074
Dryadella liliputiana	90±12	80±9	0.3014	79.61	4.9071
D. zebrine	84±7	79±7	0.2744	83.13	5.9256
Octomeria gracilis	77±7	71±5	0.2031	61.21	2.5800
Pabstiella fusca	69±8	64±7	0.1479	56.14	2.2785
Stelis grandiflora	79±5	75±7	0.2326	71.75	3.5382

Values represent means and standard deviation.

** seed volume (SV) to embryo volume (EV) ratio

Anatomical analysis

Acianthera prolifera, A. obovata, O. gracilis, and *P. fusca* all had a thin testa (0.01 µm), while the testa was thick in other species (0.03 µm). The cells of the apical pole of the embryos of *A. aphthosa*, *A. ochreata*, *A. prolifera*, and *D. lilliputiana* were smaller than those of the basal pole, while these cells were similar in size in the remaining species. Most of the species studied did not have a suspensor, which occurred only in *A. prolifera* and *D. lilliputiana* (Table 5). The presence of a cuticle surrounding the embryo was observed in *A. aphthosa* and *P. fusca*, though none of the species had a cuticle around the testa.

Table 5. Anatomical characteristics of the seed testa of the embryo cells, and presence or absence of suspensor and cuticle in species belonging to the subtribe Pleurothallidinae.

Species	Testa	Embryo cells	Suspensor	Cuticle
		Apical/basal pole		
Acianthera aphthosa	Thick	Smaller/larger	Absence	Presence/embryo
A. ochreata	Thick	Smaller/larger	Absence	Absence
A. prolifera	Thin	Smaller/larger	Presence	Presence
A. sonderiana	Thick	Similar	Absence	Absence
Anathallis obovata	Thin	Similar	Absence	Absence

Dryadella lilitipuana	Thick	Smaller/larger	Presence	Absence
D. zebrine	Thick	Smaller/larger	Absence	Absence
Octomeria gracillis	Thin	Similar	Absence	Absence
Pabstiella fusca	Thin	Similar	Absence	Presence/embryo
Stelis grandiflora	Thick	Similar	Absence	Absence

In vitro germination

The seeds of all of the micro-orchid species studied exhibited chlorophyllous embryos after three to eight days of sowing (Fig 3A). The first asymbiotic germination responses (seeds with ruptured testa and chlorophyllous protocorm) occurred after seven days of sowing for *A. prolifera* and *A. aphthosa*, and between nine and 13 days for the other species (Table 6). In general, most of the seeds had a low germination rate up to eight weeks after sowing, except for *A. prolifera* (76%). By 12 weeks after sowing, the highest germination rates were obtained for *A. prolifera*, followed by *A. ochreata* and *O. gracilis* (92%, 86%, and 77%, respectively). Of the species studied, only *A. prolifera*, *A. ochreata*, *A. aphthosa*, and *S. grandiflora* reached the seedling stage, with the time to this stage varying between four and 12 weeks (Table 6, Figs 4A and 4B).

The seeds of *A. obovata* and *D. liliputiana* showed similar behavior in the WPM, with germination rates lower than 40%, and reached the stage of protocorm with apex without the development of leaves and radicle, which became visible by 15–20 days, though they did not progress to the other stages by the 24-week evaluation. Likewise, *O. gracillis* protocorms did not develop leaves, although the germination rate was high

(77%) at 12 weeks (Table 6). The seeds of *P. fusca* had the worst germination response, with only 4% germination by eight weeks after sowing. Furthermore, the protocorms did not develop in the WPM, and they were necrotic.

Table 6. Germination rate after four, eight, and 12 weeks in woody plant medium and the average time (days) for the protocorms development of the Pleurothallidinae species.

Species	Germination* (%)			Developmental stages** (days)				
	4	8	12	I	II	III	IV	V
Acianthera aphthosa	7 b	38 c	41 cd	4	7	14	60	121
A. ochreata	35 b	53 b	86 a	4	9	14	60	119
A. prolifera	39 a	76 a	92 a	3	7	10	35	90
Anathallis obovata	12 b	23 d	36 cd	3	10	15		
Dryadella lilliputiana	4 b	15 e	38 cd	7	13	21		
Octomeria gracilis	4 b	21 d	77 b	5	11	21		
Pabstiella fusca	4 b	4 f		8	12			
Stelis grandiflora	7 b	15 e	51 c	8	11	21	95	127

* Evaluation after four, eight, and 12 weeks of sowing.

**Evaluation performed 16 weeks after sowing: I, seed with chlorophyllous embryo; II, ruptured testa (germination); III, protocorm with apex; IV, protocorm with one or two leaves; V, protocorm with leaves and root (seedling).

Means followed by the same letter in the column do not differ statistically by the Tukey test at 5% probability.

The analysis of the rates obtained at each stage of protocorm development confirmed that *A. prolifera* and *A. ochreata* had the best germination response (Fig 3). After two weeks, *A. prolifera* had the highest proportion of seeds with chlorophyll embryos (55%), followed by *A. ochreata* (52%) and *S. grandiflora* (49%) (Fig 3A). During this period, the highest germination rates (ruptured testa with chlorophyll embryo) occurred in *A. prolifera* (44%) and *A. ochreata* (39%) and were significantly higher than the other species analyzed, in which germination rates were below 10%.

By four weeks after sowing, all species except *P. fusca* had protocorms with apex (Fig 3B). By six to eight weeks after sowing, *A. prolifera* had the highest rate of protocorms with apex (27%) (Fig 3C); it was also the only species to have protocorms with leaves (21%) (Fig 3D). Seedling development began at 12 weeks, with the largest number of seedlings found in *A. prolifera* (32%), followed by *A. ochreata* (20%). The protocorms of *A. aphthosa* and *S. grandiflora* grew slowly, with less than 10% forming seedlings, though a longer period of evaluation (Fig 3E) or subculture to the same medium may be necessary. The development of the protocorms of *A. ochreata* into seedlings was somewhat slower than that of *A. prolifera*. After seedling formation, the radicles can be cut, and explants cultured in medium with cytokinins to induce shoots (Fig 4C) or explants can be subcultured for flasks with medium supplemented with activated charcoal, where elongation and root development occur (Fig 4D). Finally, *A.*

ochreata and *A. prolifera* plants were transplanted into sowing trays (3.5 cm^2) containing commercial Forth® substrates, composed of a mixture of coconut fiber, *Pinus* bark, and charcoal with fine vermiculite Eucatex® (1:1) (v/v). They were then successfully acclimatized in a greenhouse with 80% survival after three months (Fig 4E) or 12 months (Fig 4F).

Fig 3. Frequency (%) of the developmental protocorm stages of Pleurothallidinae species grown *in vitro* for two, four, six, eight, and 12 weeks in woody plant medium. Stage 1, seed with chlorophyll embryo; 2, ruptured testa (germination); 3, protocorm with apex; 4, protocorm with one or two leaves; 5, protocorm with two or more leaves and root (seedling). Means followed by the same letter do not differ statistically by the Tukey test at 1% probability.

Fig 4. *In vitro* germination of micro-orchids. (A) *Stelis grandiflora* seedling, (B-C) *Acianthera prolifera* seedling, (D) *Acianthera ochreata* before transplant, (E) *A. ochreata* after acclimatization, (F) Acclimatizated plants of *A. prolifera*. Bar: A= 0,5 cm, B-D= 1,0 cm, E= 2 cm, F= 2 cm.

Discussion

The species of the subtribe Pleurothallidinae showed considerable variation in the morphological characteristics of their seeds and embryos. The color of the seeds varied from pale yellow to brown. According to Swamy et al. [37], the microscopic size of orchid seeds makes it very difficult to visualize their color, but they are usually variations of yellow, brown, and white. Barthlott et al. [7] found that the most frequent color of seeds is brownish or dark brown, as observed in our study. Color variation may

also occur depending on the maturity of the seeds. A study found that mature seeds of *Angraecum* spp. had brown testa that was less translucent, while the immature ones were lighter and colorless [21].

We observed seeds that were ellipsoid, clavate, fusiform, or filiform, shapes that have also been reported for other orchid species [11, 15, 16, 37]. Seed shape has an evolutionary significance, with fusiform seeds found in more primitive orchids and the various other forms in more evolved epidendroid orchids [12, 40]. Fusiform seeds in *A. aphthosa*, *A. prolifera*, and *O. gracilis* were also observed in this study.

The testa in the middle part of the seed tended to be oblong or rectangular with elongated cells in the longitudinal axis of the seed [7], though *A. prolifera* cells were hexagonal and *A. hatschbachii* cells were linear. The testa cells of *A. obovata*, *A. prolifera*, and *S. grandiflora* did not show ornamentation, while papillae or verrucosities were apparent in the seeds of the other species. The presence or absence of ornamentation is cited in the literature to delimit some genera of Orchidaceae [11]. This is not the case in our study since among the species of *Acianthera*, *A. prolifera* had no ornamentation, *A. ochreata*, and *A. sonderiana* had verrucosities, and *A. aphthosa* and *A. hatschbachii* had papillae. Thus, the presence of seed ornamentation did not affect the asymbiotic germination of micro-orchids.

Variations in seed size and embryos occurred in the genera studied. In our study, *A. aphthosa* and *A. prolifera* had medium-sized seeds (500–900µm) according to the classification of [7]. Meanwhile, *A. hatschbachii*, *A. ochreata*, *A. sonderiana*, *A. obovata*, *D. zebrina*, and *O. gracilis* had small (200–500 µm) seeds, while *P. fusca* and *S. grandiflora* had very small seeds (100–200 µm) [7]. The seeds and embryos of *A. prolifera* and *A. ochreata* were larger (length, width, L/W ratio, and volume) and had higher rates of asymbiotic germination. According to Arditti et al. [2], L/W ratios

provide data on the relative degree of truncation. *Acianthera prolifera* and *A. ochreata* had L/W ratios of 2.700 and 1.767, respectively, while *P. fusca* had an L/W ratio of 0.140. Seeds with an L/W ratio of under 6.0 are referred to as truncated seeds, while those with an L/W ratio of over 6.0 are referred to as elongated seeds [3]. Based on this classification, all seeds in our study are truncated. According to Arditti et al. [2], seed volume is a better measure of seed size in orchids, which is in line with the findings of our study. They also considered that the seed volume and seed size are directly proportional to each other.

The number of testa cells along the longitudinal axis of the seed was low (2–6) in *D. liliputiana* (2), *D. zebrina* (2), *S. grandiflora* (2), *A. sonderiana* (3), *A. obovata* (4), *P. fusca* (4), *O. gracilis* (5), and *A. hatschbachii* (6). On the other hand, there was a high number (9–14) of testa cells in *A. aphthosa* (9), *A. ochreata* (10), and *A. prolifera* (14). According to Barthlott et al. [7], the number of testa cells is coupled to cell division [28], a pattern that probably arises from slow or interrupted division in the integuments following fertilization. In other genera, where cell divisions continue, the seed coats are composed of numerous small cells. Seeds with only a few cells (five or fewer) along the longitudinal axis are especially common in Orchidaceae [7].This feature is useful to characterize clades, usually at the subtribe level, with a high number of testa cells as the ancestral condition [7]. In addition, the species with a higher number of testa cells, *A. ochreata* and *A. prolifera*, showed a higher germination rate and plantlet formation in comparison with the other species analyzed.

As with the seeds, the embryos of *A. prolifera* and *A. ochreata* were also longer and wider, with greater volume. These species had higher germination rates after 12 weeks (92% and 86%, respectively). A similar result was reported by Tsutsumi et al. [38], who found that the larger embryos of *Liparis fujisanensis* F. Maek. ex Konta &

S.Matsumoto were able to germinate more rapidly than smaller embryos. According to Yeung et al. [43], orchid embryos have fewer cells and are smaller than other flowering plant embryos. This can be a result of cells having a prolonged cell cycle time. The limited number of cells produced may also be due to the early cessation of mitotic activities.

Another characteristic that varied among the species of micro-orchids was the percentage of air space between the seed coat and the embryo. The highest percentage was observed in *A. sonderiana* (87.83%) and the lowest in *O. gracilis* (61.21%) and *P. fusca* (56.14%). Güler [17] also reported that the percentage of air space in certain species of *Anacampis, Neotinea,* and *Orchis* ranged from 56 to 80%. Similar results were obtained by Swamy et al. [37], who found air space percentages ranging from 86.29% in *Cymbidium bicolor* Lindl. to 47.39% in *Coleogyne breviscapa* Lindl. Seeds with higher percentages of air space are lighter and can be more buoyant, aiding in wind dispersal across large geographic areas [4, 37]. Similarly, the seed volume to embryo volume ratio (SV/EV) was highest in *A. sonderiana* and lowest in *P. fusca*. The SV/EV ratio varied significantly among the species of the genus *Acianthera*, ranging from 8.2167 in *A. sonderiana* to 2.6729 in *A. ochreata*. This characteristic did not have any effect on the germination responses of the studied species of micro-orchids.

In our study, a suspensor was only visible in the seeds of *A. prolifera* and *D. liliputiana.* Orchidaceae is comprised of species that may present or not a suspensor [43]. Suspensor plays an important role during embryonic development, facilitating the movement of nutrients from maternal tissues to the embryo [42]. This structure may have facilitated the higher germination rates of *A. prolifera* seeds since the germination response of *D. lilliputiana* was lower. Another difference was that the seed testa of *D. lilliputiana* was thicker, and the seeds smaller than those of *A. prolifera.*

Structural polarity can occur in orchid embryos of some species, with larger cells at the apex and smaller ones at the base of the embryo, while these poles are uniform in size in other species [42]. In this study, the size of the poles was different in five species. Of these, *A. prolifera* (92%) and *A. ochreata* (86%) had high germination responses. Germination rates were lower in other three species exhibiting polarity, *A. aphthosa* (38%) and *D. liliputiana* (38%). According to Yeung [42], the cells at the apical pole of the embryo will form a meristematic zone, and the basal cells are designated to house the symbiont upon seed germination. A similar result was observed in *Phalaenopsis amabilis* (L.) Blume, which also exhibited these differences at the poles of the embryo and germinated more easily [24]. Another study found no difference between the poles of *Calypso bulbosa* (L.) Oakes., which is considered a species of difficult germination [41]. The authors found the same results for *P. fusca* and *S. grandiflora*, which had lower germination rates and developed no seedlings during the observation period. Embryos of species with no differences in size at the cell poles require additional growth and development of the embryo itself. In nature, these requirements can be provided by mycorrhizae [43].

A cuticle was present around the embryo in *A. aphthosa* and *P. fusca*. According to Aybeke [4], cuticle formation can ensure the retention of moisture by the cells of the embryo and increase its viability, which the author observed in the embryo of *Himantoglossum robertianum* (Loisel.) P.Delforge.

The results of asymbiotic germination indicated the highest germination rates in *A. prolifera* (92%), *A. ochreata* (86%), and *O. gracilis* (77%) at 12 weeks after sowing in WPM. The first two species had larger seeds and embryos with structural polarity, and both had some embryos developing to the seedlings stage in 12 to 16 weeks of cultivation. Furthermore, the lack of an endosperm may result in the seed having a

lower water holding capacity, resulting in a more rapid change in water content [43]. Meanwhile, seedlings did not develop in *O. gracilis*, despite the high germination rate, with no germinated protocorms exhibiting leaf development. Other species, such as *D. liliputiana* and *A. obovata*, had germination rates of 38% and 36%, respectively, at 12 weeks after sowing, with no protocorms forming seedlings by 24 weeks after sowing. In addition to the morphological and morphometric characteristics that varied widely among the species studied, WPM may not have provided ideal conditions for all species. Alternatively, a subculture in the WPM could be necessary to stimulate the development of protocorms in other stages. This culture medium was recommended for asymbiotic germination of *A. prolifera* (79% germination after 12 weeks), with one study finding it more effective than MS [29], MS/2 (MS with the halved concentration of salts) and KC [22] [23]. The WPM has a low concentration of salts, less total nitrogen, and less ammonium than MS [32]. According to Suzuki et al. [36], the composition of the culture medium is essential for the success of the germination of orchid seeds, with results varying significantly from one species to another. Cultivating the seeds in a medium with an adequate nutritional composition helps to increase germination and, consequently, to produce large numbers of plants, which contributes to the conservation of endangered species [36].

The species with the worst germination response was *P. fusca* (4%), the protocorms of which were all necrotic by the fourth week after sowing in the WPM. This species had the smallest seeds and embryos, a cuticle covering the embryo and no structural polarity, which may have hampered *in vitro* germination. The culture medium used may not have been appropriate for this species, or disinfesting seeds with 1% sodium hypochlorite may have had a very strong effect since the seeds had a thin testa, i.e., this process may have caused damage to the embryos. Another factor is that some

micro-orchids develop protocorms very slowly. For example, *Anathallis adenochila* (Loefgr.) F. Barros was found to take 12 months to develop seedlings (> 0.5 cm in height) [14]. In another species of the Pleurothallidinae subtribe, *Restrepia brachypus* Rchb.f., germination ranged from 7.96% in MS medium to 53.05% in Western (W) medium [27]. In addition, the protocorms of *R. brachypus* were subcultured for W medium and supplemented with banana pulp (60 g L^{-1}) for seedling development.

Another factor that may have influenced the low germination rates and slow protocorm development of some micro-orchids in our study may be the degree of seed maturity in asymbiotic germination or methods for breaking dormancy. Immature orchid seeds usually germinate better *in vitro* than ripe seeds [20], a finding that has been observed in species of the genus *Cypripedium* [44]. In contrast, Yeung et al. [43] recommend that mature seeds of epiphytic orchids must be used for asymbiotic germination. The seeds of *A. prolifera* and *A. ochreata* could have been more mature than those of *P. fusca* when they germinated *in vitro*. In addition, an appropriate composition of the culture medium is assumed to be essential for the successful germination of immature orchids [44]. Asymbiotic germination of ripe orchid seeds *in vitro* often fails due to mechanical and physiological mechanisms of dormancy. During seed maturation, the integuments become increasingly impermeable to water, or the testa and embryo may contain substances that inhibit germination even in hydrated seeds. An additional possibility is the accumulation of abscisic acid, a widespread germination inhibitor in dormant seeds, as reported by Zeng et al. [44]. Factors such as degree of maturity and dormancy of micro-orchid seeds need to be studied further for a better understanding of the germination of species that showed slow development and did not form seedlings in the WPM.

This study demonstrated considerable variation in the morphology, morphometry, and germination rates of the studied species. Germination and seedling formation of *A. prolifera* and *A. ochreata* occurred successfully in the WPM. Since germination was asynchronous, other formulations of the culture medium or supplements should be tested for their ability to accelerate the production of seedlings. Another approach would be to perform subcultures in the sowing stage so that there is no depletion of media components. Furthermore, evaluations should be carried out for more extended periods since some species grew very slowly, including *A. obovata*, *D. liliputiana*, and *P. fusca*.

Conclusions

In this study, we demonstrate efficient methods of rapid propagation of different species of Atlantic Rainforest micro-orchids. Additional studies are needed to accelerate the propagation process for some of the species that germinated slowly or minimally in this study. However, the technique used may help in the massive and rapid propagation of certain species for reintroduction into degraded habitats. Our results offer real possibilities for reintroduction programs that can play a key role in reducing the threat of extinction for species of micro-orchids.

Acknowledgments

The authors would like to thank CNPQ for the scholarship granted to Franciele Marx Koene.

References

1. Arditti J, Ghani ACA. Numerical and physical properties of orchid seeds and their biological implications. New Phytologist. 2000; 145: 367-421.
2. Arditti J, Michaud JD, Healey PL. Morphometry of orchid seeds. *I*. Paphiopedilum and native California and related species of Cypripedium. American Journal of Botany. 1979;66:1128-1137.
3. Arditti JD, Michaud JD, Healey PL. Morphometry of orchid seeds. II. Native California and related species of Calypso, Cephalanthera, Corallorhiza and Epipactis. American Journal of Botany. 1980; 67: 347-360.
4. Aybeke M. Morphological and histochemical investigations on Himantoglossum robertianum (Loisel.) P. Delforge (Orchidaceae) seeds. Plant Systematics and Evolution. 2014; 300:91-97.
5. Balilashaki K, Moradi S, Vahedi M, Khoddamzadeh AA. A molecular perspective on orchid development. The Journal of Horticultural Science and Biotechnology. 2020; https://doi.org/10.1080/14620316.2020.1727782
6. Barman D, Devadas R. Climate change on orchid population and conservation strategies: a review. Journal of Crop and Weed. 2013; 9: 1-12.
7. Barthlott W, Grobe-Veldmann B, Korotkova N. Orchid seed diversity: a scanning electron microscopy survey. In: Barthlott W, ed Englera 32 Berlin: Botanic Garden and Botanical Museum Berlin-Dahlem. 2014; 19-24.
8. BFG (The Brazil Flora Group) Growing knowledge: an overview of Seed plants diversity in Brazil. Rodriguésia. 2015; 66: 1085–1113. https://doi.org/10.1590/2175-7860201566411
9. BFG (The Brazil Flora Group) Brazilian Flora 2020: Innovation and collaboration to meet Target 1 of the Global Strategy for Plant Conservation (GSPC). Rodriguésia. 2018; 69: 1513–1527. https://doi.org/10.1590/2175-7860201869402

10. Cardoso JC, Zanello CA, Tsung Chen J. An overview of orchid protocorm-like bodies: mass propagation, biotechnology, molecular aspects, and breeding. International Journal of Molecular Sciences. 2020; 21:1-32.
11. Chase MW, Pippen JS. Seed morphology in the Oncidiinae and related subtribes (Orchidaceae). Systematic Botany. 1988; 13:313-323.
12. Dangat BT, Gurav RV. Studies on seed morphometry of Habenaria species from Western Ghats, India. Richardiana. 2016; 16:174-188.
13. Diantina S, Mcgill C, Millner J, Nadarajan J, Pritchard HW, McCormick AC. Comparative seed morphology of tropical and temperate orchid species with different growth habits. Plants. 2020; 9:161. https://doi.org/10.3390/plants9020161
14. Endres Junior D, Sasamori MH, Droste A. *In vitro* propagation of Anathallis adenochilla (Loefgr.) F. Barros (Orchidaceae), a species endemic to southern and southeastern Brazil. Acta Botanica Brasilica. 2014; 28:489-494.
15. Gamarra R, Dorda E, Scrugli A, Galan P, Ortunez E. Seed micromorphology in the genus Neotinea Rchb. f. (Orchidaceae, Orchidinae). Botanical Journal of the Linnean Society. 2007; 153:133-140.
16. Gamarra G, Ortúnez E, Cela P, Merencio A. Seed micromorphology of Orchidaceae in the Gulf of Guinea (West Tropical Africa). Plant Systematics and Evolution. 2018; 304:665-677.
17. Guller N. Seed micromorphology of Orchis Tourn. ex. L. (Orchidaceae) and allied genera growing in Edirne province, Turkey. PhytoKeys. 2016; 68:9-25.
18. Hosomi ST, Santos RB, Custodio CC, Seaton PT, Marks TR, Machado Neto NB. Pre-conditioning Cattleya seeds to improve the efficacy of the tetrazolium test for viability. Seed Science and Technology. 2011; 39:178- 189.

19. Karremans AP. Genera Pleurothallidinarum: an updated phylogenetic overview of Pleurothallidinae. Lankesteriana. 2016; 16:219 – 241.

20. Kauth PJ, Dutra D, Johnson TR, Stewart SL, Kane ME, Vendrame W. Techniques and applications of in vitro orchid seed germination. In: Teixeira da Silva JA, ed. Floriculture, ornamental and plant biotechnology: advances and topics issues**.** Isleworth: Global Science Books, 2008. pp. 375-391.

21. Kendon JP, Rajaovelona L, Sanford H, Fang R, Bell J, Sarasan V. Collecting near mature and immature orchid seeds for ex situ conservation:‘in vitro collecting' as a case study. Botanical Studies. 2017; 58:1-14.

22. Knudson, L. A new nutrient solution for the germination of orchid seed. American Orchid Society Bulletin. 1946; 14: 214-217.

23. Koene FM, Amano E, Ribas LLF. Asymbiotic seed germination and in vitro seedling development of Acianthera prolifera (Orchidaceae). South African Journal of Botany. 2019; 121:83-91.

24. Lee YI, Yeung EC, Lee N, Chung MC. Embryology of Phalaenopsis amabilis var. formosa: embryo development. Botanical Studies. 2008; 49:139–146.

25. Lloyd G, McCown BH. Commercially-feasible micropropagation of mountain laurel, Kalmia latifolia, by use of shoot-tip culture. Proceedings of the International Plant Propagators' Society. 1980; 30:421-427.

26. Menini Neto L, Docha Neto A. Redescoberta e tipificação de Octomeria leptophylla Barb. Rodr. (Orchidaceae), micro-orquídea endêmica de Minas Gerais, Brasil. Rodriguesia. 2009; 60:461-465.

27. Millner HJ, Obeng A, McCrea AR, Baldwin TC. Axenic seed germination and *in vitro* seedling development of Restrepia brachypus (Orchidaceae). The Journal of the Torrey Botanical Society. 2008;135:497-505.

28. Molvray M, Chase MW 2003. Seed morphology. In: Pridgeon AM, Cribb PJ, Chase MW, Rasmussen FN, eds. Genera Orchidacearum Volume 1. General Introduction, Apostasioideae, Cypripedioideae New York: Oxford University Press.

29. Murashige, T., Skoog, F. A revised medium for rapid growth and bioassays with tobacco tissue culture. Physiologia Plantarum. 1962; 15, 473-497.

30. Myers N, Mittermeyer RA, Mittermeier CG, Fonseca GAB, Kent J. Biodiversity hotspots for conservation priorities. Nature. 2000; 403:853-858.

31. Pence V. The possibilities and challenges of in vitro methods for plant conservation. Kew Bulletin. 2010; 65:539-547.

32. Phillips GC, Garcia M.. Plant tissue culture and practices. In Vitro Cellular and Development Biology – Plant. 2019; 55:242-257.

33. Pridgeon AM, Blanco M, Gomes RS, Grayer R, Hermans J, Stenzel H, Veith NC. Subtribe Pleurothallidinae. In: Pridgeon AM, Cribb PJ, Chase MW, Rasmussen FN, eds. Genera Orchidacearum. New York: Oxford University Press, 2005. pp. 319-422.

34. Pridgeon AM, Cribb PJ, Chase MW, Rasmussen FN. Genera Orchidacearum. Oxford: University Press, 1999.

35. Simonelli M, Fraga CN. Espécies da flora ameaçadas de extinção no estado do Espírito Santo. Vitoria: Ipema, 2007.

36. Suzuki RM, Moreira VC, Pescador R, Ferreira WM. Asymbiotic seed germination and *in vitro* seedling development of the threatened orchid Hoffmannseggella cinnabarina. In Vitro Cellular e Development Biology – Plant. 2012; 48: 500-511.

37. Swamy KK, Kumar HK, Ramakrishna TM, Ramaswamy SN. Studies on seed morphometry of epiphytic orchids from Western Ghats of Karnataka. Taiwania-Taipei. 2004; 49:124-140.

38. Tsutsumi C, Yukawa T, Lee NS, Lee CS, Kato M. Phylogeny and comparative seed morphology of epiphytic and terrestrial species of Liparis (Orchidaceae) in Japan. Journal of Plant Research. 2007; 120:405-412.

39. Vij SP, Pathak P. Orchid diversity: conservation and utilization. Proceedings of the National Academy of Sciences, India Section B: Biological Sciences. 2012; 82: 295-300.

40. Vij SP, Pathak P, Kaur P, Sharma M. Somatic/artificial seeds in orchids. Orchid News. 1992; 8:12-13.

41. Yeung EC, Law SK. Embryology of Calypso bulbosa. II. Embryo development. Canadian Journal of Botany. 1992; 70:461-468.

42. Yeung EC. A perspective on orchid seed and protocorm development. Botanical Studies. 2017; 58:1-14.

43. Yeung EC, Li YY, Lee YI. Understanding seed and protocorm development in orchids. Lee YY and Yeung EC (eds.) Orchid Propagation: from laboratories to greenhouses—methods and protocols. Springer Protocols Handbooks, 2018. pp 1-24 https://doi.org/10.1007/978-1-4939-7771-0_1

44. Zeng S, Zhang Y, Teixeira da Silva J.A, Wu K, Zhang J, Duan J. Seed biology and in vitro seed germination of Cypripedium. Critical Reviews in Biotechnology. 2014; 34: 358-371.

Click here to access/download;Figure;fig1.tif.tiff

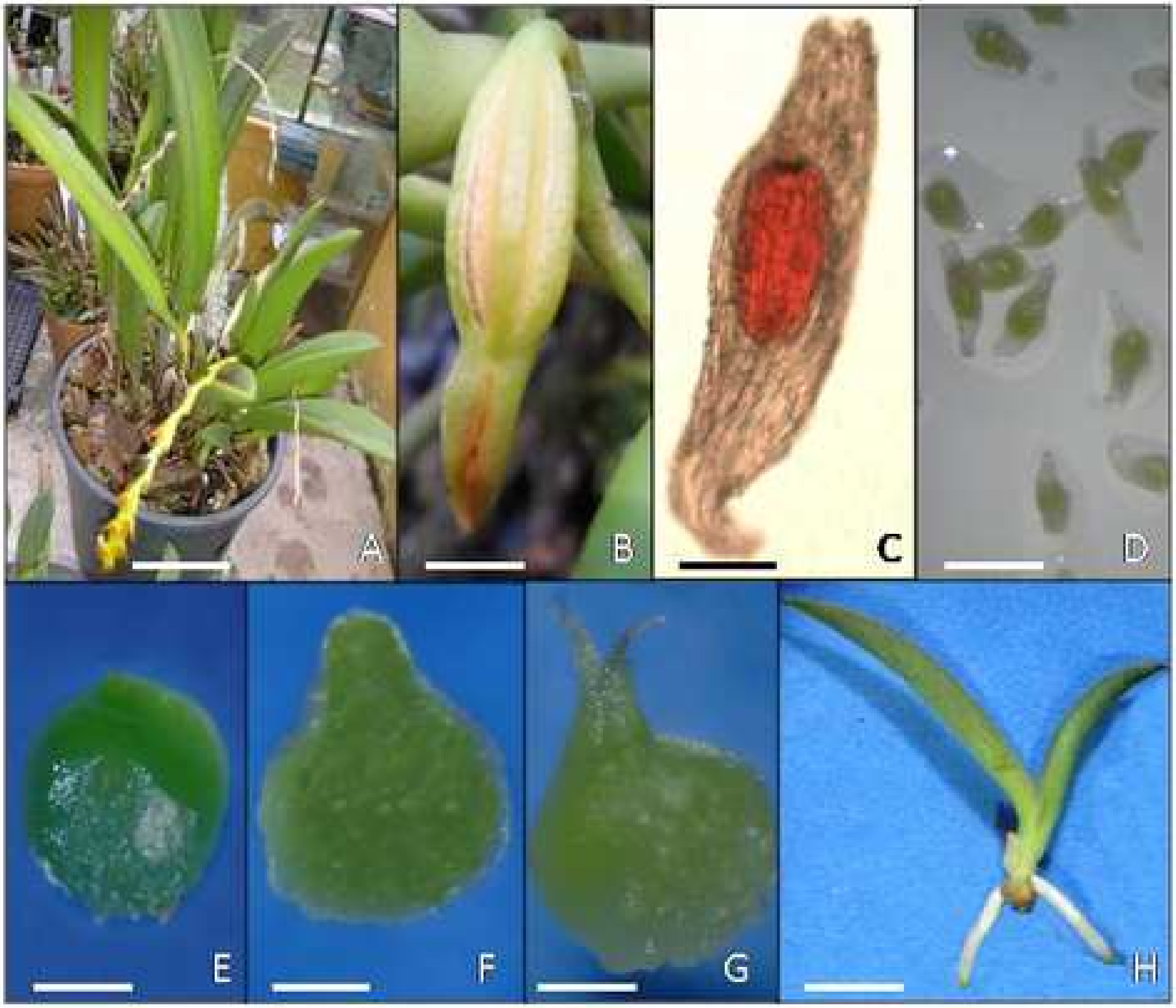

Fig2.tif
Click here to access/download;Figure;fig2.tif.tif

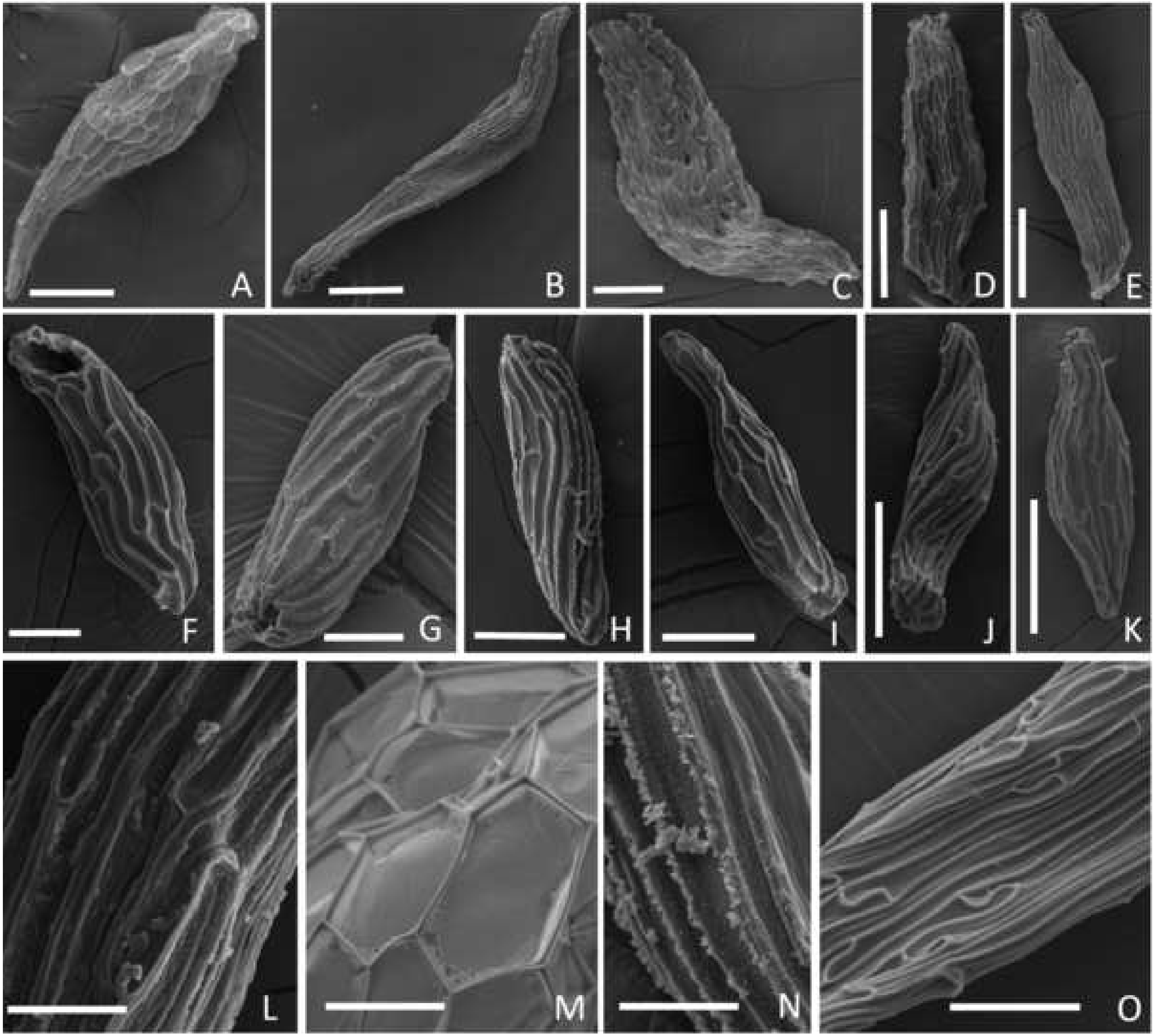

Sem t^tulo.tif

Fig3.tif

Click here to access/download;Figure;fig3.tif.tiff

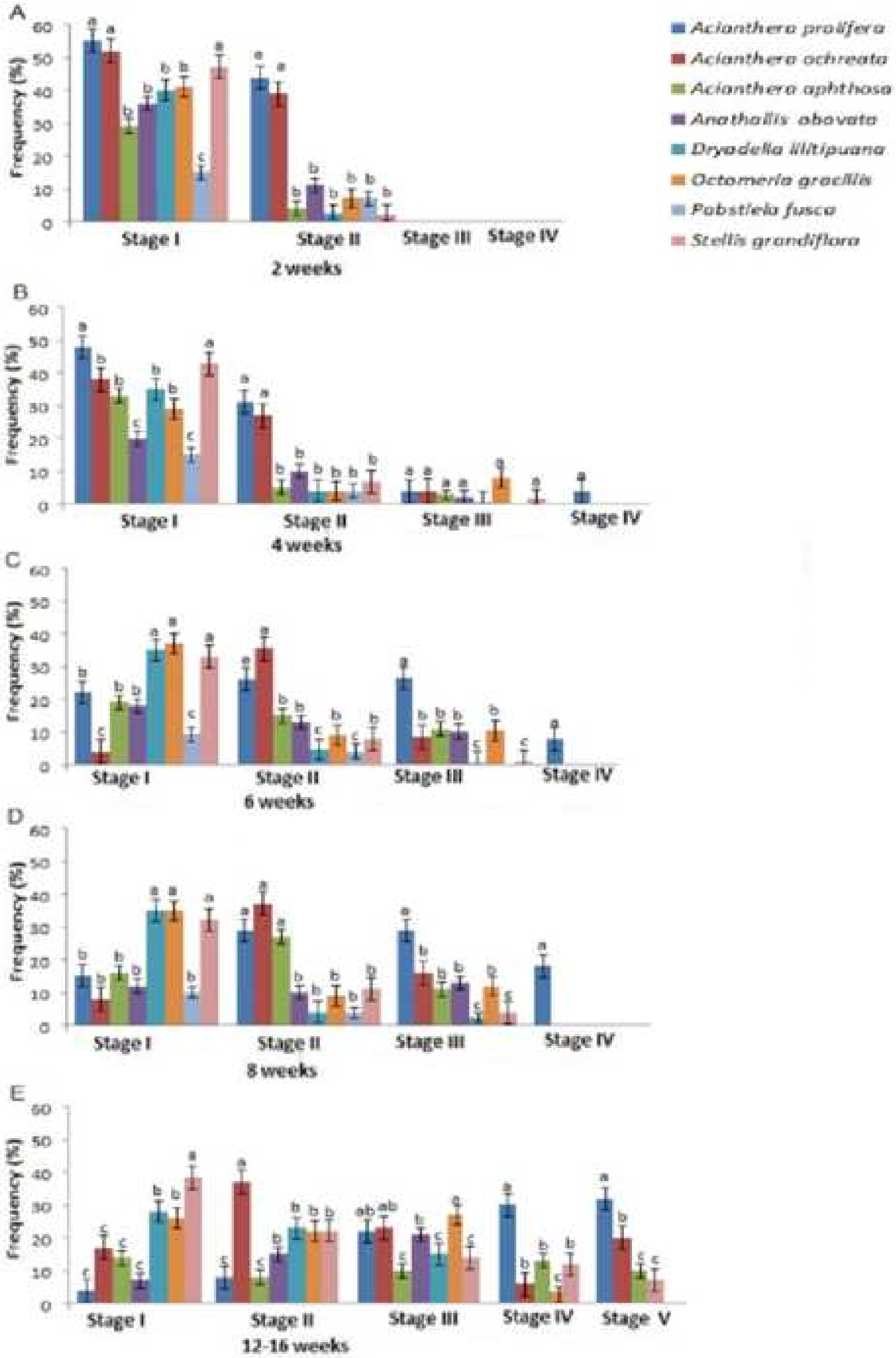

 Click here to access/download;Figure;Fig 4.tif.tiff

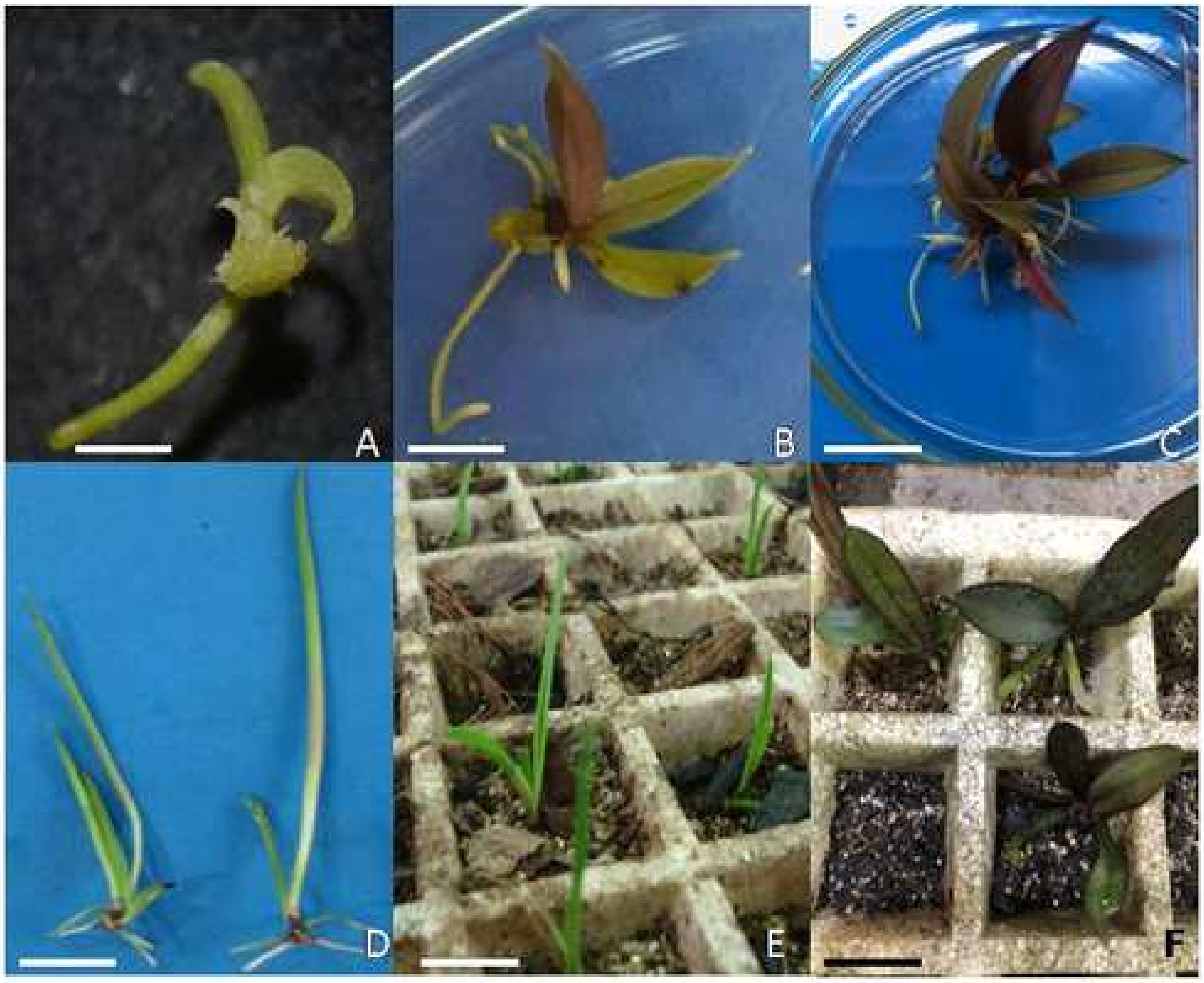

authors contributions

Click here to access/download
Supporting Information
author contributions.docx
