# Supplementary material for: Asymbiotic germination and morphological studies of seeds of Atlantic Rainforest micro-orchids (Pleurothallidinae)
Source: PLoS One. 2020 Dec 14;15(12):e0243297. doi: 10.1371/journal.pone.0243297 (PMC7735581; doi:10.1371/journal.pone.0243297)
Supplement: Attachment — Submitted filename: Review- PONE-D-20-27111.docx [file pone.0243297.s001.docx]

**PLOS ONE**

**Manuscript Number:** PONE-D-20-27111

**Title:** A symbiotic germination and morphological studies of seeds of Atlantic Rainforest micro-orchids (Pleurothallidinae)

**Article Type: Research Paper**

***Mona soliman***

**Review Report**

The initial research articles in PLOS ONE describes the goals and the theories, methodologies and results of research and analysis of primary and unpublished studies. Original study papers can also include validation studies and disconfirmation of results that allow the exclusion and/or reproducibility of previously published findings by hypothetical assumptions. The manuscript entitled “**A symbiotic germination and morphological studies of seeds of Atlantic Rainforest micro-orchids (Pleurothallidinae)”** is interesting and mentioned to efficient methods of rapid propagation of different species of Atlantic Rainforest micro-orchids.

**I recommend some minor changes before the paper is accepted in this periodical.**

**Abstract**

Abstract is well written and informative

**Introduction**

The introduction is clearly and constructively written

**Materials and Method**

**Line 123-132:**

References are needed of all the above qualitative and quantitative methods

**Line 141-149:**

You must be subtitled to this paragraph to note the implications that the reader needs to understand……….thanks for your effort

**Line 161-166:**

In the results chapter, add the figures and tables to the correct place.

**Results**

These are nicely written

**Discussion**

The whole chapter deals with its purpose. The parameter studies are explained logically, but I think there should be a small reduction in discussions as far as possible

**Comments for editor**

Thank you for taking my manuscript review into consideration

I just need to check the paper again, as it is good, and only need minor corrections to be published and made available to readers.
