# Supplementary material for: Asymbiotic germination and morphological studies of seeds of Atlantic Rainforest micro-orchids (Pleurothallidinae)
Source: PLoS One. 2020 Dec 14;15(12):e0243297. doi: 10.1371/journal.pone.0243297 (PMC7735581; doi:10.1371/journal.pone.0243297)
Supplement: Attachment — Submitted filename: reviwers responses.docx [file pone.0243297.s003.docx]

To Dr: Jen-Tsung Chen
Academic Editor of PLOS ONE

Dear Dr. Jen-Tsung Chen

We greatly appreciate all the comments and suggestions from reviewers that have enriched the quality of our work. We are forwarding a version of the manuscript with the corrections suggested by the three reviewers. Corrections in blue are for reviewer 1, in green for reviewer 2 and in red for the reviewer 3. We have carefully considered and adopted almost all suggestions in our revised manuscript (PONE-D-20-27111) entitled: ***Asymbiotic germination and morphological studies of seeds of Atlantic Rainforest micro-orchids (Pleurothallidinae)*.** Point-by-point responses to the reviewers’ comments are shown below, with our responses shown in italic font.

Hoping for a favorable reply.

Yours sincerely,

Luciana Lopes Fortes Ribas

Department of Botany

Federal University of Paraná

Curitiba, Brazil

e-mail: llfribas@ufpr.br

**Responses to Reviewer 1**

**1.** In Abstract: Add few starts lines about the background information of the research

**Answer:** *Your request was accepted, see lines* ***25-28***

**2.** Add some most relevant keywords in the manuscript

**Answer:** *Relevant keywords have been added in blue*

***Keywords:*** *Orchidaceae; micromorphology;* ***morphometry****, in vitro germination; woody plant medium;* ***SEM.***

**3.** Revise the reference format as per journal author guidelines.

**Answer:** *The references format was reviewed and corrected as guidelines of Plos One*

**Responses to Reviewer 3**

Materials and Method

**1.** Line 123-132:

References are needed of all the above qualitative and quantitative methods
**Answer**: *The references were added,* ***see lines 154-156***

**2.** Line 141-149:
You must be subtitled to this paragraph to note the implications that the reader needs to understand……….thanks for your effort

**Answer**: *Your request was accepted,* ***see lines*** ***159 and 166***

**3.** Line 161-166:
In the results chapter, add the figures and tables to the correct place.

**Answer**: *This has been corrected in the manuscript.*

**4. Discussion**

The whole chapter deals with its purpose. The parameter studies are explained logically, but I think there should be a small reduction in discussions as far as possible

**Answer**: *Your request was accepted.*

**5-** About statystical analysis there are a mistake in comparisons of different species in Table 6. This comparison is similar to compare the natality of 'cats and lyons'. Also, comparisons between sizes of 'cats and lyons' is not biologically interesting. Also, a description about how many plants were used for measurements were not provided. In addition, the authors needs clarify if the time of evaluation was considered as treatment in the analysis. Tukey tests are not sound interesting in these cases, but only the standard deviation could be used for these analysis.

**Answer**: *Statistical analysis was removed from the table 6.*

**Responses to Reviewer 4 (J. C. Cardoso)**

**Abstract**

**1**. WPM medium is not a conventional medium for orchid germination. Authors have some reference support the use of this culture for this purpose? Because germination of orchids depends strong from the mineral composition.

References and the justification for using this medium were included in the manuscript.

**Answer:** *WPM medium was previously tested for Acianthera prolifera and compared with other formulations (MS, MS/2 and KC) and showed better response, accelerating seedling development, so it was used to evaluate the germination of the other 10 micro-orchid species. It was also recommended for other species of orchids, such as: Brasiliorchis picta and Hadrolaelia grandis when compared to conventional medium such as: MS, MS/2, KC and VW.*

*The references are below and the justification for using the WPM medium were placed on the*

*lines* ***64-71 and 83-85*** *in the manuscript*

*Koene FM, Amano E, Ribas LLF. Asymbiotic seed germination and in vitro seedling development of Acianthera prolifera (Orchidaceae). South African Journal of Botany. 2019; 121:83-91.*

*Santos SA, Smidt EC, Padial AA, Ribas LLF. Asymbiotic seed germination and in vitro propagation of Brasiliorchis picta. African Journal of Biotechnology. 2016; 15:134–144.*

*Vudala SM, Ribas LLF. Seed storage and asymbiotic germination of Hadrolaelia grandis (Orchidaceae). South African Journal of Botany. 2017; 108:1–7.*

**2-** What authors considering seedlings?

**Answer:** *In our study is the stage that corresponds to protocorms with two or more leaves and roots* ***(See Line 182****). Some authors use plantlet for this stage. Seedlings are plants that develop from the embryo of a seed.*

**3-** “The protocorms of *Anathalis obovata*, *Dryadella liliputiana*, and *Octomeria gracillis* developed slowly in the WPM, not reaching the seedling stage in 24 weeks of cultivation”. Is this a problem of culture media use or from the species?

**Answer:** *These species were tested only in this medium. To be sure if it is a problem of the medium or species, studies are needed comparing various formulations of culture medium or a subculture in the WPM could be necessary. This was also discussed in the lines* ***460-467.***

*The two studies on seed germination of the subtribe Pleurothallidinae species, reported percentage of germination at most 50% testing various formulations of culture medium and slow development of protocorms, requiring a subculture for the formation of seedlings.*

**4-** “This morphological and morphometric study contributes to the understanding of asymbiotic germination of some micro-orchid species” **Please explain how?**

**Answer:** *The seeds and embryos of the species that germinated faster and with high percentages (A. prolifera and A. ochreata) presented some characteristics, such as: larger size and seed volume, structural polarity, higher number of testa cells and this may have contributed to this response. In our study the species with the lowest germination response was P. fusca (4%), this species had the smallest seeds and embryos, a cuticle covering the embryo and no structural polarity. We also observed that the percentage of air space between the seed coat and the embryo did not have effect on the germination responses of the studied species of micro-orchids. Further studies with other genera and species of the Subtribe Pleurothallidinae, and other culture media are needed to assess the influence of these morphological and morphometric characteristics, however, we achieved high germination percentages after six months of cultivation in WPM medium.*

**Introduction**

**5-** Please the references follow numerical order, not alphabetic order. Please correct.

**Answer:** *This has been corrected.*

**6- ...** their high germination rates, which are commonly over 70%, as opposed to under 5% in ex vitro conditions**.** This is highly dependent on the species. Terrestrial orchids in general have many difficulties for germination using asymbiotic germination, compared with symbiotic.

**Answer:** *It was clarified in the text that this statement is for epiphytic species at* ***line 62.*** *Discussion with species of terrestrial orchids was also excluded.*

**7- What the reason for choice eleven species and these is specifical? Occurrence in region?**

**Collection?**

**Answer:** *These species were previously pollinated and were selected for having mature capsules at the time of our study. Our goal was to compare the largest number of micro-orchid species, but we depended on the formation of capsules.*

**Material and methods**

**6- These species are uncommon! I recommended that the authors add a figure with the eleven species, if possible, at the reproductive stage.**

**Answer:** *A figure with the studied species was added as requested.*

**5- How was realized the pollination? Self-pollination? Crossing handling? Naturally?**

**If naturally, there are no possible hybridation?**

**Answer:** *Each species had three to four plants in a greenhouse, in which manual cross-pollination was performed.*

**6-** How was the identification of species? Comparisons between the vouchers at herbarium?

**Answer:** *The species were identified by comparisons between the vouchers at herbarium and with the help of the specialist in this Subtribe (Pleurothallidinae): Eric de Camargo Smidt (co-author) of this manuscript.*

**7-** Recently legislation of Brazilian flora requires the registration of wild species in SISGEN system. Authors have these numbers for wild species collected in Brazil?

**Answer:** *This registration is not required to studies with no accessions to the genes content or associated knowledge. Also, these plants came from outside Natural Protected Areas; they grow in periurban areas of Curitiba or purchased from reputable companies that produce and sell the species.*

**Results**

**8- 80% of survival for 3 months. For what species? In what environmental conditions? Please describe.**

**Answer:** *80% of survival after three months for A. prolifera and A. ochreata. The environmental conditions in greenhouse were complemented. See lines* ***187-195*** *and* ***509-516.***

**Conclusions**

**Answer:** *They were corrected.*

**Figures 3 and 4**

**Very low quality figures**

**Answer:** *The figures were reviewed and prepared according to the guidelines of Plos One*
